# Trends in Abundance of Sea Lice *Lepeophtheirus salmonis* and *Caligus clemensi* on Juvenile Wild Pacific Salmon Unchanged Following Cessation of Salmon Aquaculture in Coastal British Columbia

**DOI:** 10.1111/jfd.14136

**Published:** 2025-04-22

**Authors:** Simon R. M. Jones, Crawford W. Revie, Lance Stewardson

**Affiliations:** ^1^ Fisheries and Oceans Canada Pacific Biological Station Nanaimo British Columbia Canada; ^2^ Department of Computer and Information Sciences University of Strathclyde Glasgow Scotland UK; ^3^ Mainstream Biological Consulting Campbell River British Columbia Canada

**Keywords:** *Caligus clemensi*, *Lepeophtheirus salmonis*, salmon aquaculture, sea lice, wild salmon

1

Phased removals of net‐pen salmon aquaculture facilities since 2020 in the Discovery Islands (DI) and other coastal regions of British Columbia (BC), Canada, are in response to concerns that salmon aquaculture adversely impacts the conservation of wild salmon. Harm to locally migrating juvenile wild salmon caused by elevated risk of exposure to sea lice derived from infestations on the cultured salmon is one hypothesized impact mechanism (Morton and Williams 2003; Krkošek et al. 2007; Krkošek and Hilborn 2011). The salmon louse *Lepeophtheirus salmonis* and the herring louse *Caligus clemensi* are reported from cultured Atlantic salmon *Salmo salar* and from wild Pacific salmon *Oncorhynchus* spp. In BC, although cultured salmon are sources of sea lice infestations (Jeong et al. 2025), the relative magnitude of these sources is uncertain because of a paucity of information on the natural infestations on reservoir species. Given the specificity for *L. salmonis* to mature on salmonids, resident over‐wintering Pacific salmon are candidate reservoirs (Beamish et al. 2005). Alternatively, Pacific herring *Clupea pallasi* and threespine stickleback *Gasterosteus aculeatus* are reservoirs of *C. clemensi* (Jones et al. 2006; Beamish et al. 2009). The removal of Atlantic salmon production has provided an opportunity to observe natural sea lice infestations in these regions.

In the DI region, the removal of Atlantic salmon aquaculture facilities began in 2021 and since 2022 the productivity has been zero. Two Chinook salmon facilities remain operational with limited production and have been inactive since the fall of 2022. A statistically significant reduction in sea lice abundance reported on juvenile chum salmon *Oncorhynchus keta* and pink salmon *O. gorbuscha* from the DI between 2020 and 2022 was attributed to this decline in production (Routledge and Morton 2023). The purpose of the present study was to provide the first description of sea lice infestations on juvenile wild salmon in the DI over a time span that included both the presence and absence of active Atlantic salmon aquaculture facilities from 2017 to 2024.

Beach seine procedures for sample collection were adapted from the Canadian Department of Fisheries and Oceans (DFO) in the Broughton Archipelago (Hargreaves et al. 2004). Up to 30 individual fish per species per site were collected in both months. Individual fish were haphazardly recovered from the net and packaged separately in re‐sealable bags labelled with the site name, date and sample number. Bagged fish were euthanized immediately after capture by freezing with dry ice.

Laboratory analysis of frozen samples was conducted by the BC Centre for Aquatic Health Sciences (BC‐CAHS, Campbell River, BC). Each fish was partially thawed, identified to species, and the weight and fork length measured. All sea lice observed during microscopic examination of the fish were identified to species and to developmental stage: copepodid, chalimus, pre‐adult (male or female) or adult (male or female) (Kabata 1972; Hamre et al. 2013). Sea lice infestations were described using prevalence (proportion of sample infested) and mean abundance (number of lice per number of fish examined).

Atlantic salmon production in metric tonnes (MT) at each of the 15 aquaculture facilities operating and potentially contributing to the sea lice infestation pressure within the study area during the wild salmon migration was estimated from the total closing biomass at the end of May during each study year. Biomass at the two active Atlantic salmon facilities adjacent to the study area (Figure 1) was not included.

**FIGURE 1:**
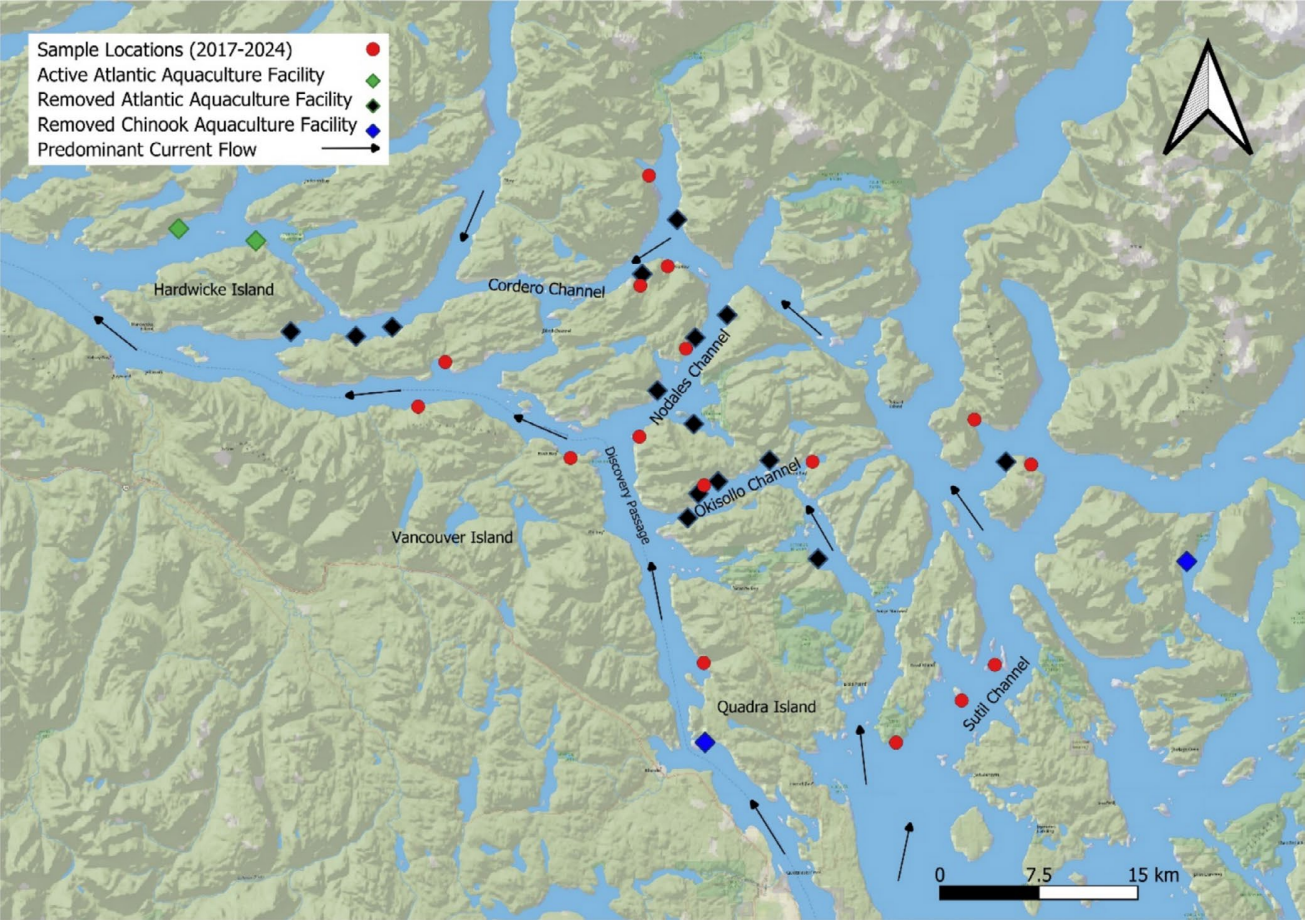
The Discovery Islands, British Columbia, showing locations of 16 beach seine sites used between 2017 and 2024, and the predominant current flow through the Islands. The locations of the 15 Atlantic salmon farms that were removed between 2020 and 2022, together with the two Chinook salmon farms, are also shown.

A total of 5967 juvenile salmon from the DI were examined for sea lice between 2017 and 2024 (Table 1). The mean weight of chum salmon ranged from 0.89 g (2017) to 1.79 g (2019) and of pink salmon from 0.33 g (2023) to 1.22 g (2024). The prevalence of all sea lice infestations on chum salmon ranged from 2.9% (2023) to 26.9% (2019) and on pink salmon from 0% (2023) to 17.1% (2020) (Figure 2). The mean abundance of all lice on chum salmon ranged from 0.04 (2023) to 0.39 (2019) and on pink salmon from 0 (2023) to 0.30 (2020) (Table 1). The overall proportion of *L. salmonis* as a percentage of all sea lice on chum salmon was 57.3% (25.0%–74.2%) and 44.2% on pink salmon (22.7%–61.4%) (Table 2). No sea lice of either species were detected on pink salmon in 2023.

**TABLE 1:** Mean abundance (±SE) of *Lepeophtheirus salmonis* (Lep), *Caligus clemensi* (Cal) and both infestations (Total) on juvenile chum salmon ( *Oncorhynchus keta* ) and pink salmon ( *Oncorhynchus gorbuscha* ) collected from the Discovery Islands region of coastal British Columbia between 2017 and 2024.

Year	Chum				Pink			
	*N*	Lep	Cal	Total	*N*	Lep	Cal	Total
2017	622	0.04 ± 0.01	0.05 ± 0.01	0.09 ± 0.01	198	0.06 ± 0.02	0.08 ± 0.02	0.13 ± 0.03
2018	405	0.04 ± 0.01	0.02 ± 0.01	0.06 ± 0.01	259	0.04 ± 0.01	0.08 ± 0.02	0.12 ± 0.02
2019	405	0.29 ± 0.03	0.10 ± 0.02	0.39 ± 0.04	324	0.07 ± 0.01	0.07 ± 0.01	0.11 ± 0.02
2020	359	0.24 ± 0.04	0.08 ± 0.02	0.32 ± 0.05	339	0.18 ± 0.03	0.12 ± 0.02	0.30 ± 0.04
2021	504	0.06 ± 0.01	0.02 ± 0.01	0.14 ± 0.02	426	0.06 ± 0.01	0.12 ± 0.02	0.18 ± 0.02
2022	519	0.03 ± 0.01	0.09 ± 0.02	0.12 ± 0.02	343	0.03 ± 0.01	0.10 ± 0.02	0.13 ± 0.02
2023	309	0.02 ± 0.01	0.02 ± 0.01	0.04 ± 0.01	278	0.00 ± 0.00	0.00 ± 0.00	0.00 ± 0.00
2024	358	0.06 ± 0.01	0.09 ± 0.02	0.15 ± 0.02	319	0.07 ± 0.02	0.11 ± 0.02	0.18 ± 0.03
Total	3481				2486			

**FIGURE 2:**
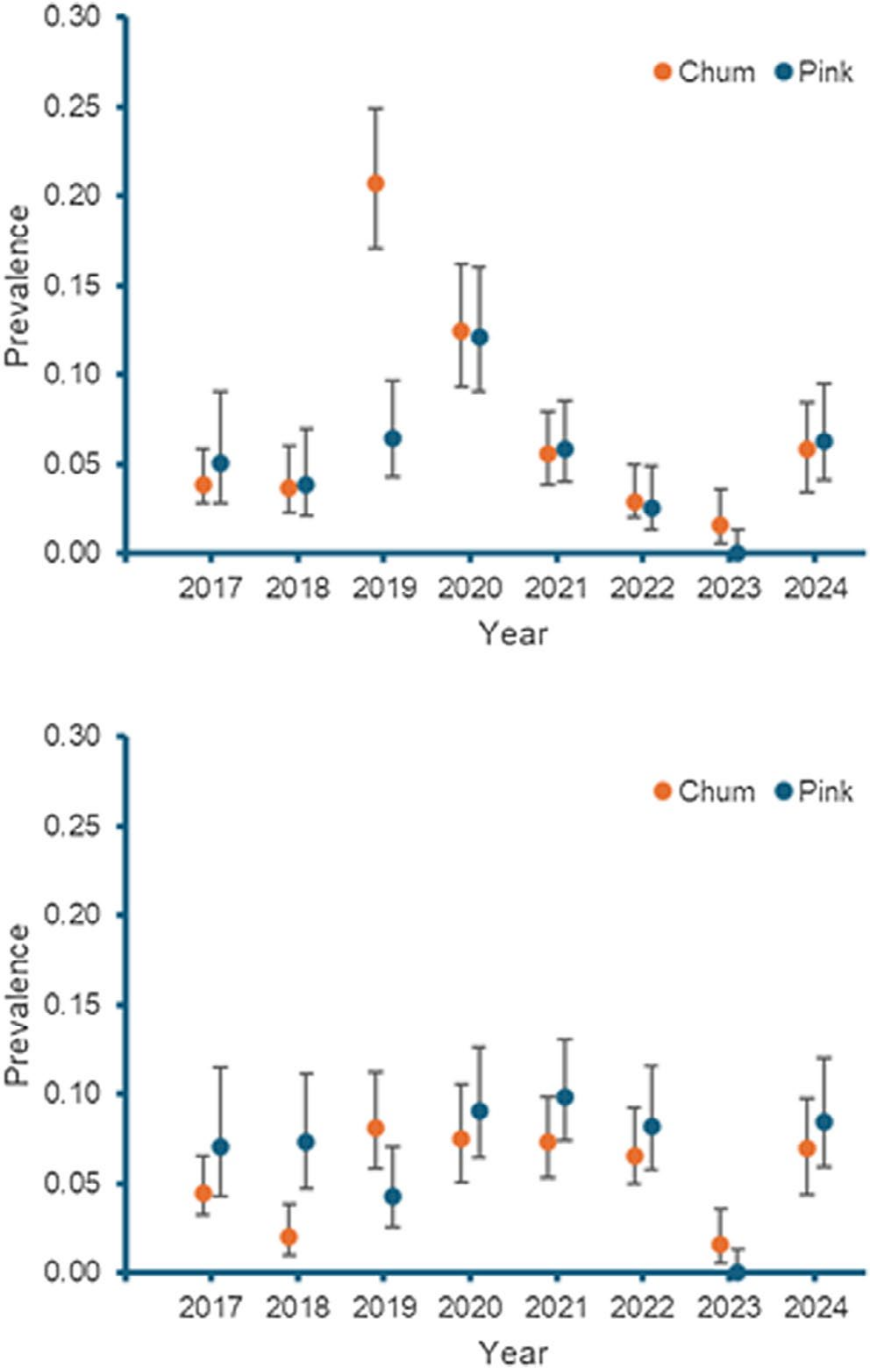
Prevalence (95% CI) of infestations with *Lepeophtheirus salmonis* (top) and *Caligus clemensi* (bottom) on chum salmon ( *Oncorhynchus keta* ) and pink salmon ( *Oncorhynchus gorbuscha* ) from the Discovery Islands coastal region of British Columbia between 2017 and 2024.

**TABLE 2:** Relative proportions of *L. salmonis* (Lep) and *C. clemensi* (Cal) on chum salmon ( *O. keta* ) and pink salmon ( *O. gorbuscha* ) in the Discovery Islands coastal region of British Columbia between 2017 and 2024. Estimated biomass in metric tonnes from cultured Atlantic salmon at open net‐pen facilities in the study area.

Year	Chum			Pink			Atlantic aquaculture facilities	
	Total lice	% Lep	% Cal	Total lice	% Lep	% Cal	Number active	Biomass (MT)
2017	56	44.6	55.4	26	42.3	57.7	6	8080
2018	23	65.2	34.8	32	34.4	65.6	7	12,037
2019	159	74.2	25.8	37	59.5	40.5	6	10,895
2020	115	73.9	26.1	101	61.4	38.6	9	18,126
2021	70	40.0	60.0	75	34.7	65.3	2	4086
2022	60	25.0	75.0	44	22.7	77.3	0	0
2023	11	54.5	45.5	0	—	—	0	0
2024	54	40.7	59.3	58	39.7	60.3	0	0
Total	548	57.3	42.7	373	44.2	55.8		

A key finding of this study was the persistence of sea lice infestations on juvenile chum salmon and pink salmon in the DI despite the absence of Atlantic salmon aquaculture production between 2022 and 2024. Previously, a 96% decline in sea lice abundance on juvenile chum salmon and pink salmon from the DI between 2020 and 2022 was attributed to the removal of salmon aquaculture in the region (Routledge and Morton 2023). The present study revealed a similar reduction in the prevalence and abundance of *L. salmonis* between 2020 and 2022 both on chum salmon and pink salmon, a trend which continued in 2023 and was also seen in *C. clemensi* in that year. However, in the continued absence of salmon aquaculture in 2024, the prevalence and mean abundance increased on pink salmon to levels greater than measured between 2017 and 2019, and on chum salmon to levels greater than measured in 2017 and 2018. Similar trends were also evident among infestation levels of *L. salmonis* or *C. clemensi* individually, emphasising the roles of environmental factors and indicating that the declines observed between 2020 and 2023 were not entirely dependent on reductions in aquaculture productivity. The northwesterly migration of juvenile salmon through the study area combined with the predominantly northwesterly flow of seawater in this region (Foreman et al. 2015) reduces the likelihood that the adjacent active facilities contributed to the observed infestations on the wild salmon. The prevalence of sea lice on juvenile salmon in the adjacent Broughton Archipelago coastal region during a similar decline in aquaculture productivity, was also depressed in 2023 relative to 2022 and 2024 (Jones et al. unpublished manuscript), further reinforcing the role of environmental factors and the need to better characterise them [Correction added on 13 May 2025 after first online publication: The publication year for the reference “Jones et al. 2025” has been corrected to “Jones et al. unpublished manuscript” in this version.].

The risk of exposure to infective sea lice copepodids is a function of the magnitude of the source infestation combined with the modulating effects of temperature, salinity, and distance (Jeong et al. 2025; Brooker et al. 2018; Brooks 2005). The aquaculture biomass reported in the present study did not appear to adequately account for the likely source infestation magnitude. This study provides additional rationale for improved environmental monitoring as a tool in predicting impacts to the conservation of juvenile wild salmon.

## Author Contributions

**Simon R. M. Jones:** writing – original draft, writing – review and editing, formal analysis, validation, methodology. **Crawford W. Revie:** writing – review and editing, writing – original draft, formal analysis, validation, methodology. **Lance Stewardson:** conceptualization, writing – original draft, writing – review and editing, formal analysis, data curation, methodology, project administration.

## Conflicts of Interest

Lance Stewardson (a Director of Mainstream Biological Consulting) has an ongoing annual contract with the aquaculture producers in BC (Cermaq Canada, Grieg Seafood BC and MOWI Canada West) to sample wild juvenile salmon for sea lice infestation assessment to fulfil the conditions of the licence or as part of environmental certification programmes. The other authors declare no conflicts of interest.

## Data Availability

The data that support the findings of this study are available from the corresponding author upon reasonable request.
